# sox4 And sox11 Function during *Xenopus laevis* Eye Development

**DOI:** 10.1371/journal.pone.0069372

**Published:** 2013-07-18

**Authors:** Wiebke Cizelsky, Annemarie Hempel, Marlen Metzig, Si Tao, Thomas Hollemann, Michael Kühl, Susanne J. Kühl

**Affiliations:** 1 Institute for Biochemistry and Molecular Biology, Ulm University, Ulm, Germany; 2 International Graduate School in Molecular Medicine Ulm, Ulm University, Ulm, Germany; 3 Institute for Physiological Chemistry, Martin-Luther-University Halle-Wittenberg, Halle/Saale, Germany; Indiana University, United States of America

## Abstract

*SoxC* genes are involved in many developmental processes such as cardiac, lymphoid, and bone development. The *SoxC* gene family is represented by *Sox4*, *Sox11,* and *Sox12*. Loss of either Sox4 or Sox11 function is lethal during mouse embryogenesis. Here, we demonstrate that *sox4* and *sox11* are strongly expressed in the developing eye, heart as well as brain in *Xenopus laevis*. Morpholino oligonucleotide mediated knock-down approaches in anterior neural tissue revealed that interference with either Sox4 or Sox11 function affects eye development. A detailed analysis demonstrated strong effects on eye size and retinal lamination. Neural induction was unaffected upon Sox4 or Sox11 MO injection and early eye field differentiation and cell proliferation were only mildly affected. Depletion of both genes, however, led independently to a significant increase in cell apoptosis in the eye. In summary, Sox4 and Sox11 are required for *Xenopus* visual system development.

## Introduction

In *Xenopus,* eye development starts with the induction of the eye field in the anterior neural plate at the end of gastrulation. The eye anlage is characterized by the expression of eye specific marker genes such as *rax*, *pax6,* and *six3*. Loss of any of these genes leads to strong defects during early eye development [1]. In contrast, the overexpression of these factors can result in the formation of ectopic eyes [1]. During neurulation, the single eye field splits into two eye anlagen located on either side of the embryo, a process supported by the underlying prechordal mesoderm [2]. At the end of neurulation, two optic vesicles evaginate from the neural tube at the level of the prospective diencephalon towards the overlaying ectoderm. Through the contact of the eye vesicle with the epidermis, the lens placode is thickened in the epidermis. Later, the distal half of the eye vesicles invaginates into the embryo thereby forming a bilayered optic cup.

The optic cup can further be subdivided into the thinner outer retinal pigmented epithelium (RPE) and the thicker neural retina in which six major neuronal cell types and glial cells develop. In the mature retina, different well structured layers can be distinguished: the outer nuclear layer (ONL) containing the cell bodies of cone and rod photoreceptors, the inner nuclear cell layer (INL) including the cell bodies of bipolar, horizontal, and amacrine interneurons, and the ganglion cell layer (GCL) containing the cell bodies of ganglion cells. Muller glial cells span all retinal layers.

The founding member of the *Sox* (sry-related box) gene superfamily of transcription factors was the male sex determination gene *Sry* (sex determining region Y). *Sox* proteins contain a single high mobility group (HMG) domain, which is involved in DNA binding. This domain of all *Sox* gene family members has an identity of more than 50% to the HMG box of the *Sry* gene. Currently, 20 *SOX* genes in human and mouse are known which are classified into ten groups, *SoxA* to *SoxJ,* based on sequence similarity and similar DNA binding properties [3]. Through protein-protein interactions, Sox proteins are also able to recruit additional proteins to DNA. They can act either as transcriptional activators or repressors depending on the cellular context and the interaction partners [4]. During development, Sox proteins are essential for many processes such as the regulation of pluripotency, gastrulation, differentiation, and organogenesis [5]–[7].

Sox4, Sox11, and Sox12 form the SoxC protein family. It has been shown that members of the SoxC family reveal overlapping expression and are functionally redundant [7]–[9]. In some tissues however, they differ in expression levels and transactivation rates [9]. During mouse development, *SoxC* genes show a widespread, largely overlapping expression pattern with highest levels in post-mitotic neuronal progenitor cells of the neural tube, the dorsal root ganglia, the thalamus, the retina, and the cerebral and cerebellar cortex. In addition, transcripts are found in undifferentiated mesenchymal cells, the genital tubercle, endocardial cushions of the heart, the lung, the gut, the pancreas, and the nephrogenic mesenchyme [7]. The knock-out of either *Sox4* or *Sox11* in mice is lethal at E14 or directly after birth, respectively, due to severe cardiac defects such as outflow tract malformations. In addition, these mice display further developmental defects including abnormalities in lymphocyte development (Sox4), or in eye and bone development (Sox11) (reviewed in [7]). In contrast, *Sox12* null mice are viable and show no obvious malformations [9].

The expression and function of soxC genes during *Xenopus laevis* embryogenesis has not been investigated so far. Here we describe for the first time the spatiotemporal expression profile of *sox4 and sox11* during early *Xenopus* development in detail. Functional analyses using specific morpholino oligonucleotides (MOs) targeting either *sox4* or *sox11* demonstrated a requirement of both genes during eye development, in particular for the formation and lamination of the retina. In Sox4 or Sox11 depleted eyes, cell apoptosis was significantly induced whereas cell proliferation was not affected. Our data indicate an important role for Sox4 and Sox11 during vertebrate visual system development.

## Results

### *Sox4* and *sox11* are Expressed in the Developing Eye of *Xenopus laevis*

A search in the GenBank database revealed a full-length cDNA sequence of the *Xenopus laevis sox4* gene (Acc. No. NM_001098441). Based on this sequence, we successfully cloned a *Xenopus laevis sox4* cDNA. Sequencing of several independent clones consistently revealed one amino acid exchange at position 180 from asparagine to serine in comparison to the published sequence and thus was considered to represent a polymorphism. Cloning of *sox11* was already described [10].

**Figure 1:**
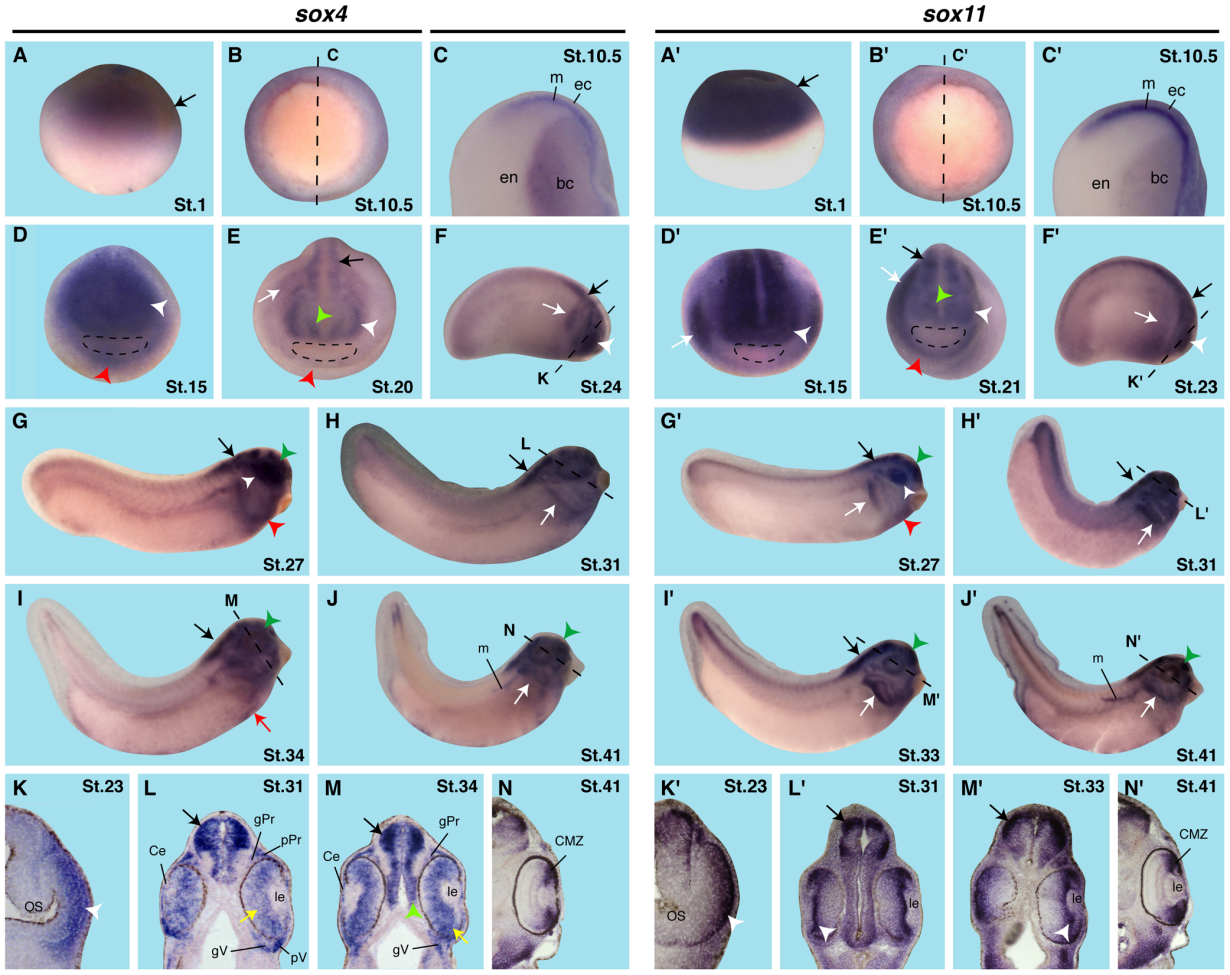
Spatial expression of *sox4* and *sox11* during *Xenopus laevis* embryogenesis. **A–N** **** *sox4* expression, **A’–N’** *sox11* expression. The cement gland is depicted by a dotted circle. **A,A’:** At stage 1, *sox4* and *sox11* are maternally expressed at the animal pole (arrows). **B,B’:** During gastrulation, *sox4* and *sox11* are expressed in the expanding mesoderm surrounding the blastoporus (arrows). Dotter lines indicate the level of sections shown in C,C’. **C,C’:** Sections through embryos at stage 10.5 indicate a strong expression in the mesoderm (m). Ectoderm (ec) and endoderm (en) are negative for *sox4* and *sox11*. bc = blastocoel. **D,D’:** At stage 15, *sox4* and *sox11* are strongly expressed in the anterior neural tissue (white arrowheads). In addition, *sox4* transcripts are visualized in the cardiac progenitors (red arrowhead) whereas sox11 is detected at the placodal primordium (white arrow). **E,E’:** At stage 20, *sox4* and *sox11* are expressed in the neural tube (black arrows), the forebrain (green arrowheads), the eye anlagen (white arrowheads), the migrating neural crest cells (white arrows) and cardiac progenitor cells (red arrowheads). **F,F’:** *Sox4* and *sox11* mRNA molecules are detected in the neural tube (black arrows), the eye (white arrowheads) and the migrating neural crest cells (white arrows). The dotted line indicates the level of section shown in K and K’. **G,G’:** At stage 27, *sox4* and *sox11* are expressed in the hindbrain (black arrows), the eye (white arrowheads), the forebrain (green arrowheads), and the cardiac region (red arrowheads). **H,H’:** *Sox4* and *sox11* are strongly expressed in the hindbrain (black arrows) and branchial arches (white arrows). The dotted line indicates the level of section shown in L and L’. **I,I’:** *Sox4* and *sox11* transcripts are visualized in the hind- and forebrain (black and green arrows) as well as branchial arches (white arrows). *Sox4* is additionally visualized in the vitelline veins (red arrow). Dotted line indicates the level of section shown in M and M’. **J,J’:** At stage 41, *sox4* and *sox11* are expressed in the head mesenchyme (white arrows), the forebrain (green arrowheads) and migratory primordium of the lateral line system (m). **K–N,K’–N’:** Transverse sections at indicated stages. **K,K’:** *Sox4* and *sox11* are detected in the optic vesicle (white arrowheads). Note that expression is missing from the optic stalk (os). **L:** *Sox4* transcripts are detected in the retina (yellow arrow), the cornea epithelium (Ce) and the hindbrain (black arrow). In addition, *sox4* is expressed in the placodes and the ganglia of the profundal (pPr; gPr) and trigeminus (pV; gV) nerve as well. Note that the placodal expression is only in the deep layers whereas the superficial layer is quite free of staining. **L’:** *Sox11* expression is detected in the brain (black arrow), strongly in the ganglion cell layer of the retina (white arrowhead) with a gradient towards outer retinal layers. **M:** *Sox4* transcripts are visualized in the retina (yellow arrow), the corneal epithelium (Ce), the mid- (black arrow) and forebrain (green arrowhead). A strong expression in the profundal (gPr) and trigeminus (gV) ganglia is shown. **M’:** *Sox11* mRNA is visualized in the differentiated dorsal interneurons of the neural tube (black arrow) and the retina with a strong expression in the ganglion cell layer (white arrowhead). **N, N’:** *Sox4* and *sox11* are expressed in the ciliary marginal zone (CMZ) of the eye.

We first determined the spatiotemporal expression of the three *soxC* members during early *Xenopus* embryogenesis. As we did not detect any significant expression of *sox12* in the eye, we subsequently focused on *sox4* and *sox11* during early *Xenopus* embryogenesis. For this purpose, we generated *antisense* RNA probes that bind to the open reading frame of either *sox4* or *sox11* endogenous mRNA. Open reading frames of *sox4* and *sox11* revealed a nucleotide sequence homology of only 57%. Thus, we considered the probes to be specific for either sox4 or sox11. For detailed tissue-specific expression, we also generated vibratome sections of stained embryos (Fig. 1).

In the early *Xenopus* embryo, *sox4* and *sox11* were detected in the animal pole region of the embryo (Fig. 1A,A’). During gastrulation, *sox4* and *sox11* mRNA molecules were strongly visualized in mesodermal cells (Fig. 1B, B’,C,C’) and at stage 15 in the anterior neural plate (Fig. 1D,D’). *sox4* was additionally detected in the cardiac progenitor cell population (CPCs; Fig1D) and *sox11* in the placodal primordium (Fig. 1D’). During late neurulation and tailbud stages, both *sox* transcripts were expressed in different brain regions, the posterior neural tube, the eye vesicles, the migrating neural crest cells, and the CPCs (Fig. 1E–H,K,E’–H’,K’). During tadpole stages, *sox4* was expressed in the first and the second heart field lineages, the vitelline veins and the aortic arch arteries (Fig. S1). At stage 34, sections revealed a strong *sox4* expression in the meso- and pericardium, and more faintly in the pericardial roof, the endo- and myocardium of the closed heart tube (Fig. S1). Sections through the head region of tadpole *Xenopus* embryos demonstrated that *sox4* transcripts were located in the retina and the cornea epithelium of the eye as well as in defined regions of the brain (Fig. 1L,M). At stage 41, *sox4* was expressed in the ciliary marginal zone (CMZ) of the eye (Fig. 1N).

*Sox11* expression in the retina was strongly detectable in the ganglion cell layer forming a gradient towards more outer retina layers (Fig. 1L’,M’), which is different to *sox4* expression (compare to Fig.1L,M). At stage 41, *sox11* expression was found in the CMZ identical to *sox4* (Fig.1N’).

In summary, *sox4* and *sox11* are strongly expressed in the developing *Xenopus* eye with an overlapping expression pattern suggesting a role for both genes during early eye development.

### Sox4 and Sox11 are required for *Xenopus* Eye Development

Since *sox4* and *sox11* are specifically expressed in the *Xenopus* eye, we aimed to study the function of both genes during *Xenopus* visual system development by performing loss of function experiments using an *antisense* morpholino oligonucleotide (MO) based approach. For this purpose, we designed a MO targeting the translation start site of the endogenous *sox4* mRNA (Fig. S2A). To investigate Sox11 function, we used a previously described Sox11 MO [10]. For rescue experiments, full-length human SOX4 or *Xenopus sox11* constructs were injected which are not targeted by Sox4 MO (Fig. S2B) or Sox11 MO [10], respectively.

**Figure 2:**
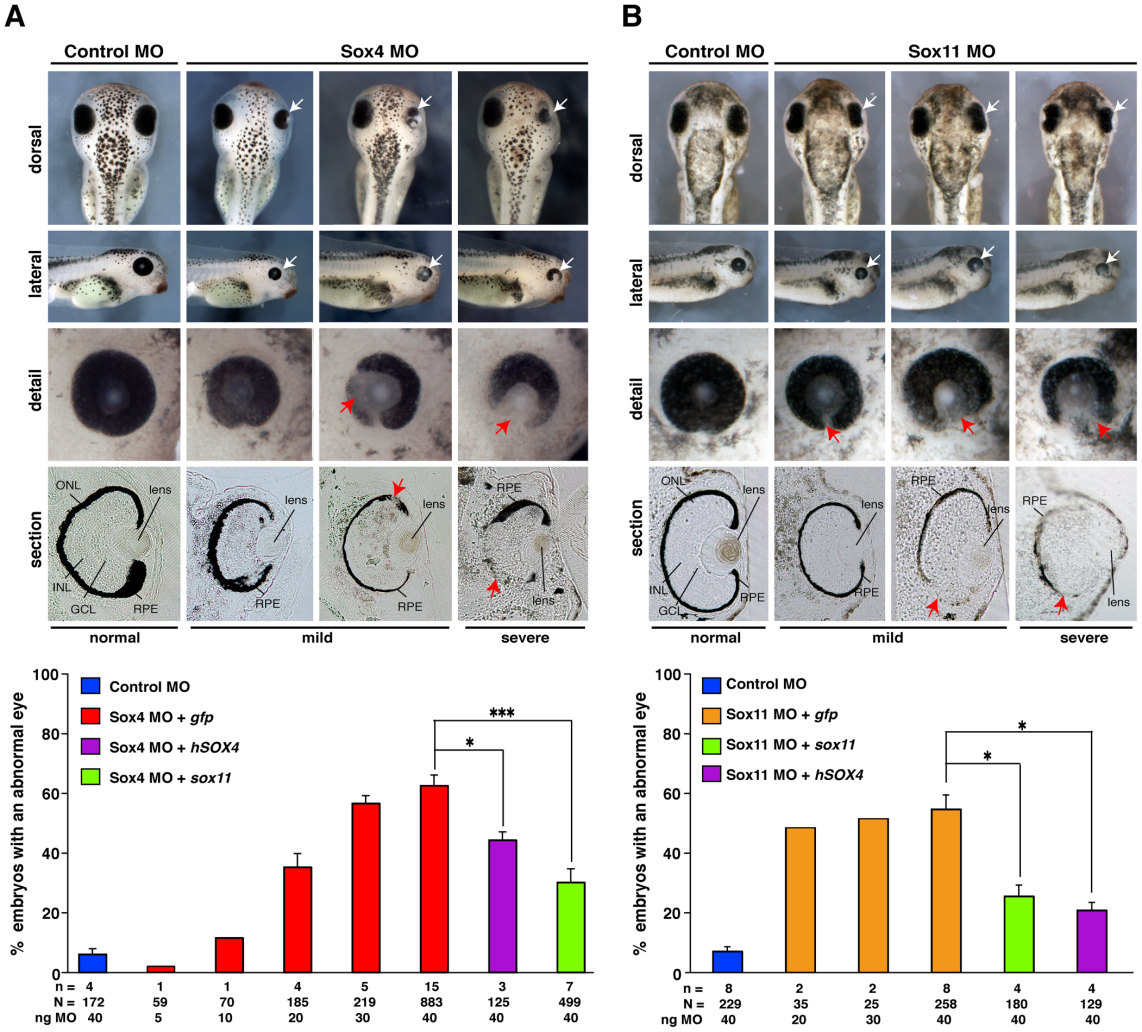
Sox4 or Sox11 loss of function results in an abnormal eye development. Effect of Sox4 (A) and Sox11 (B) depletion on eye development. The injection of Sox4 or Sox11 MO leads to smaller, deformed, and severely deformed eyes (white arrows) as illustrated in a dose dependent manner. A Control MO injection had no effect on eye development. Detailed views demonstrated defects in the formation of the RPE (red arrows). Vibratome sections showed that Control MO injection had no influence on the formation of the retinal layers and the RPE as well whereas depletion of Sox4 or Sox11 resulted in an abnormal RPE and retinal layering (red arrows). Quantitative representations are given. The eye phenotype of Sox4 or Sox11 down-regulation was significantly rescued by the co-injection of either *SOX4* or *sox11* RNA. GCL = ganglion cell layer, INL = inner nuclear cell layer, n = number of independent experiments, N = number of injected embryos analyzed, ONL = outer nuclear cell layer, RPE = retinal pigment epithelium. Error bars indicate standard error of the means (s.e.m.), * P≤0.05, *** P≤0.001.

To test whether Sox4 or Sox11 have an influence on eye development, we injected several doses of both MOs into *Xenopus* embryos, fixed and investigated them at stage 41 when the retinal pigmented epithelium (RPE) is visible. Intriguingly, loss of either gene led to a quite similar eye phenotype. Depletion of Sox4 or Sox11 resulted in strong eye defects including smaller and deformed eyes whereas the uninjected side as well as the Control MO injected embryos developed normally (Fig. 2). Additionally, the RPE was not completely developed. Vibratome sections revealed severely disorganized retinal lamination upon Sox4 or Sox11 deficiency what we investigated in more detail using specific retinal marker genes (see below). To examine the specificity of the used MOs, we co-injected one of the MOs together with human *SOX4* or *Xenopus sox11* RNAs as mentioned above. Indeed, human *SOX4* was able to significantly restore the eye defect induced upon loss of either Sox4 or Sox11 (Fig. 2 and Fig. S3). In addition, *sox11* RNA was also able to revert loss of Sox11 as well as Sox4 function. These experiments clearly demonstrate the specificity of the MOs used and suggest that both genes are functionally redundant during eye development. Injection of Sox4 and Sox11 MOs together using low MO doses however did not reveal a cooperative effect of both genes (data not shown).

Taken together, these data indicate a requirement of Sox4 and Sox11 during *Xenopus* eye development.

### Sox4 and Sox11 Affect Retinal Lamination

**Figure 3:**
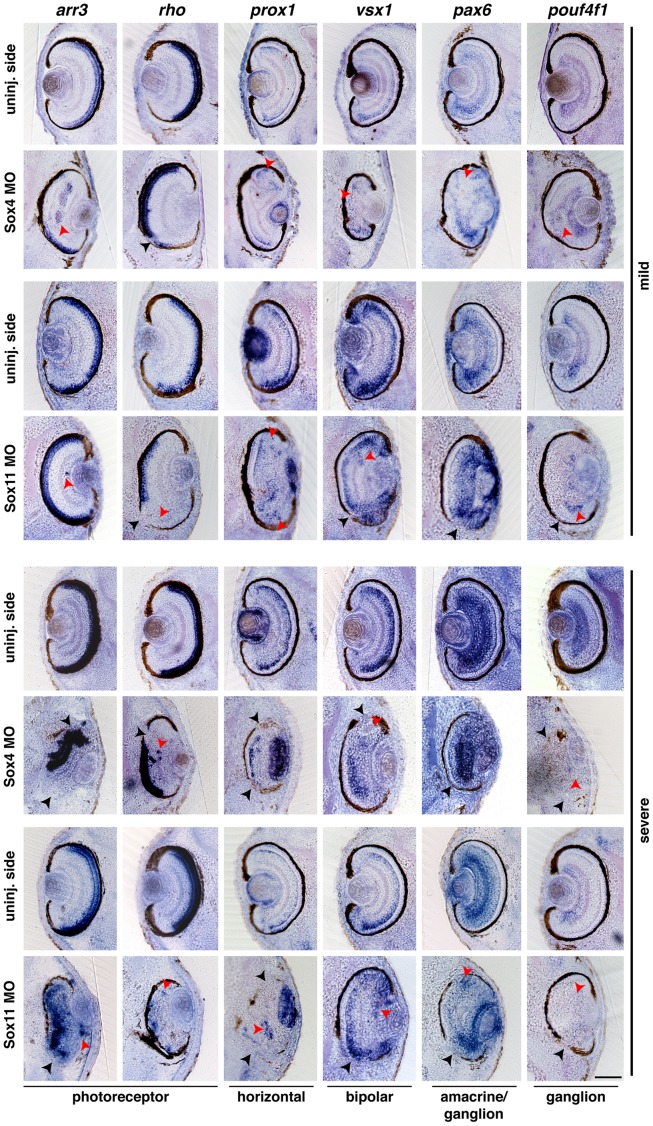
Sox4 depletion interferes with retinal lamination. Unilateral injection of 40 ng Sox4 or 40 ng Sox11 MO had no effect on the primary formation of most retinal cell types as shown by the expression of specific marker genes in mild eye phenotypes at stage 41. Only ganglion cells disappeared upon depletion of Sox4 or Sox11 in the severe eye phenotype. Many retinal cells were displaced (red arrowheads). Especially photoreceptor cells are displaced into inner layers of the retina (red arrowheads). In addition, the RPE is affected (black arrowheads). The uninjected (uninj.) sides revealed normal retinal lamination. For each marker gene, several embryos of different independent experiments were analyzed and showed a similar phenotype. Scale bar indicates 100 µm.

Since *sox4* and *sox11* are specifically expressed in the developing retina (Fig. 1L,M,L’,M’) and the depletion of both genes lead to severe eye defects including disarranged retinal layers (Fig. 2), we raised the question whether the formation of the different retinal cell types was affected upon Sox4 or Sox11down-regulation. To examine these different cell types, we performed whole mount *in situ* hybridization experiments (WMISH) using embryos at stage 41 with mild and severe eye phenotypes (Fig. 2) and probes for well characterized retinal cell specific marker genes [11]. Subsequently, we performed vibratome sections of stained embryos. We used *arr3* and *rho* [12] to determine photoreceptor cells, *pax6* [13] to visualize amacrine and ganglion cells, *vsx1* [14] to detect bipolar cells, *prox1* [15] to show horizontal cells, and *pouf4f1* [16] to stain for ganglion cells. Almost all analyzed marker genes were expressed in the mildly and severely affected eyes. Only *pouf4f1* was absent in the severely affected ones (Fig. 3). These data indicate that all retinal cell types were formed excluding ganglion cells. Intriguingly, we observed a severe disorganization of the different retinal layers as indicated by ectopic and missing expression domains of marker genes (Fig. 3, red arrowheads). Furthermore bipolar and amacrine cells are displaced into the inner or outer layers of the retina (red arrowheads).

**Figure 4:**
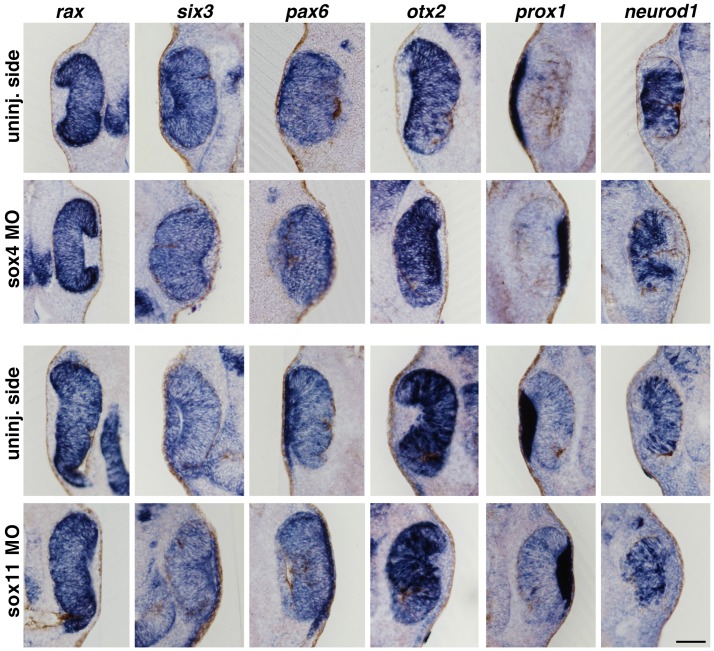
Proneural genes are not affected upon loss of Sox4 or Sox11. At stage 32, expression of proneural genes was not changed after loss of Sox4 or Sox11. Scale bar indicates 100 µm.

To analyze when this phenotype becomes apparent during development, we furthermore examined the same set of marker genes at stage 36 when the retina cell types start to differentiate. We observed a similar phenotype at this stage (data not shown). Next, we investigated the expression of the proneural genes *rax*, *six3*, *pax6*, *otx2*, *prox1,* and *neurod1* at stage 32 before retinal cells are differentiated [17]. At this earlier stage, we could not detect a change in gene expression indicating that Sox4 and Sox11 have no influence on generating neuronal progenitor cells in the retina (Fig. 4).

In summary, Sox4 or Sox11 depletion had an influence on the fate of ganglion cell type and resulted in severely disturbed retinal lamination.

### Sox4 and Sox11 in Lens Development

**Figure 5:**
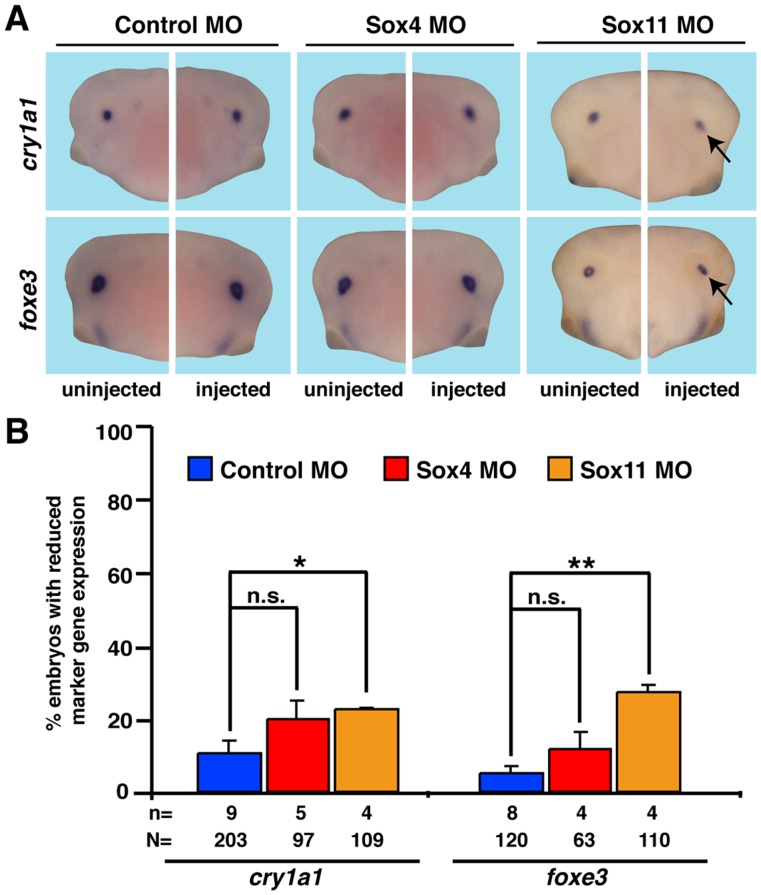
Lens development upon Sox4 or Sox11 depletion. Lens development was normal in Sox4 depleted embryos as shown by marker gene expression at stage 36. Loss of Sox11 resulted in a mild change of marker gene expression. n = number of independent experiments, N = number of injected embryos analyzed, n.s. = not significant, * P≤0.05,** P≤0.01.

To investigate lens formation upon Sox4 or Sox11 depletion, we injected both MOs independently and examined the expression of the two lens marker genes *cyr1a1* and *foxe3* at stage 36 (Fig. 5). As expected from *sox4* expression pattern in the eye (Fig. 1), loss of Sox4 had no effect on the expression of both genes while Sox11 depletion had a mild effect on both lens markers.

### Sox4 and Sox11 in Early Eye Field Induction and Eye Differentiation

**Figure 6:**
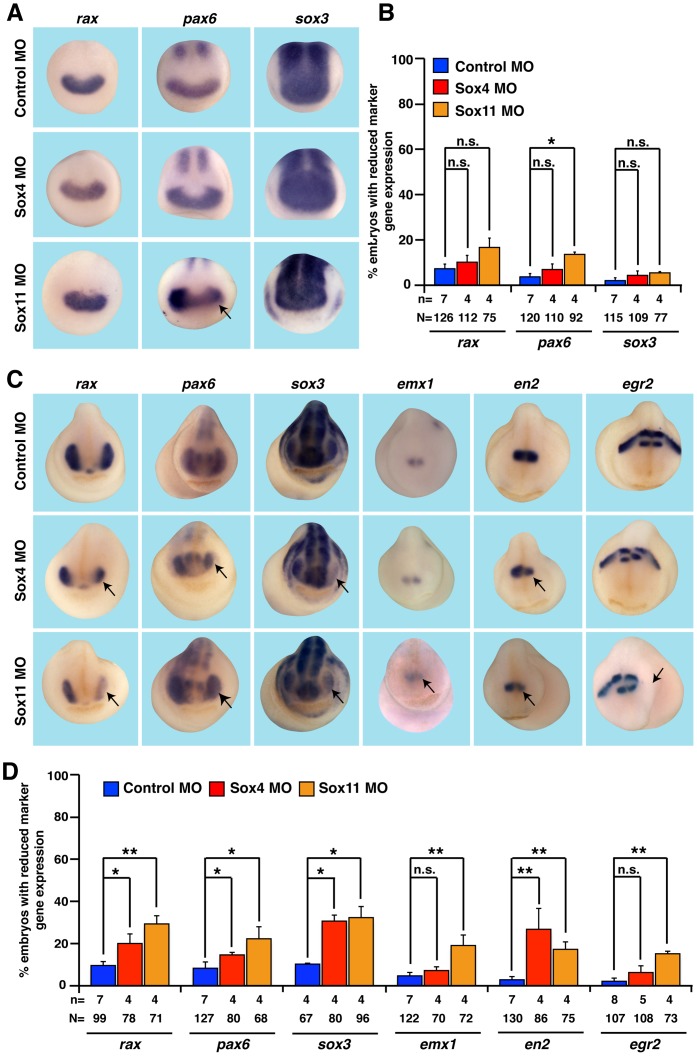
Eye-specific marker genes are unaffected upon loss of Sox4 or Sox11 function. **A and B:** Depletion of Sox4 function had no influence on marker gene expression at stage 13. Loss of Sox11 led to a slight decrease of *pax6* expression. **C and D:** At stage 23, Sox4 depleted embryos revealed a reduced expression of *rax, pax6, sox3* and en2. *Emx1* and *egr2* expression was not affected. In contrast, all marker genes were mildly affected upon Sox11 knock-down. **B and D.** Quantitative representations are given. n = number of independent experiments, N = number of injected embryos analyzed, n.s. = not significant, * P≤0.05,** P≤0.01.

Beside the strong effect on retinal lamination, loss of either Sox4 or Sox11 function also led to the formation of smaller eyes in comparison to the uninjected side or Control MO injected embryos (Fig. 2). A possible reason for this phenomenon could be a defect in eye induction or early eye differentiation. Thus, we investigated the expression of the pan-neural marker gene *sox3* as well as *rax* and *pax6* as marker genes for the eye field at stage 13 in Sox4 or Sox11 depleted embryos. Neither Sox4 nor Sox11 function was required for the expression of *sox3* and *rax* (Fig. 6A). Sox11 depletion led to a mild down-regulation of *pax6* in the eye field. Moreover, we examined *rax, pax6* and *sox3* expression at stage 23 and observed a reduction in gene expression in the eye region in some Sox4 or Sox11 depleted embryos (Fig. 6B). Since *sox4* and *sox11* are also expressed in brain and neural crest cells, we tested the expression of *emx1* (marker gene for the forebrain), *en2* (marker gene for the isthmus), and *egr2* (marker gene for specific regions of the hindbrain and migrating neural crest cells). Whereas all three analyzed genes showed a mild reduction upon loss of sox11 function, only *en2* was reduced upon sox4 knock-down (Fig. 6B). In all cases however, the phenotype had a low penetrance.

Taken together, we conclude that neural induction is not affected and early differentiation of neural tissue is only mildly affected upon Sox4 or Sox11 suppression. Defects in early neural specification can therefore be excluded as the main reason for the severe late eye phenotype.

### Sox4 and Sox11 in Regulation of Cell Proliferation and Apoptosis

To clarify whether the smaller eyes observed upon Sox4 or Sox11 depletion was due to a misregulation of cell proliferation or apoptosis, we performed corresponding assays at stage 23, 32, and 41 using Sox4 or Sox11 deficient embryos. At stage 23, we could not observe any change in cell proliferation or apoptosis upon sox4 depletion compared to the uninjected side or control MO injected embryos (data not shown).

**Figure 7:**
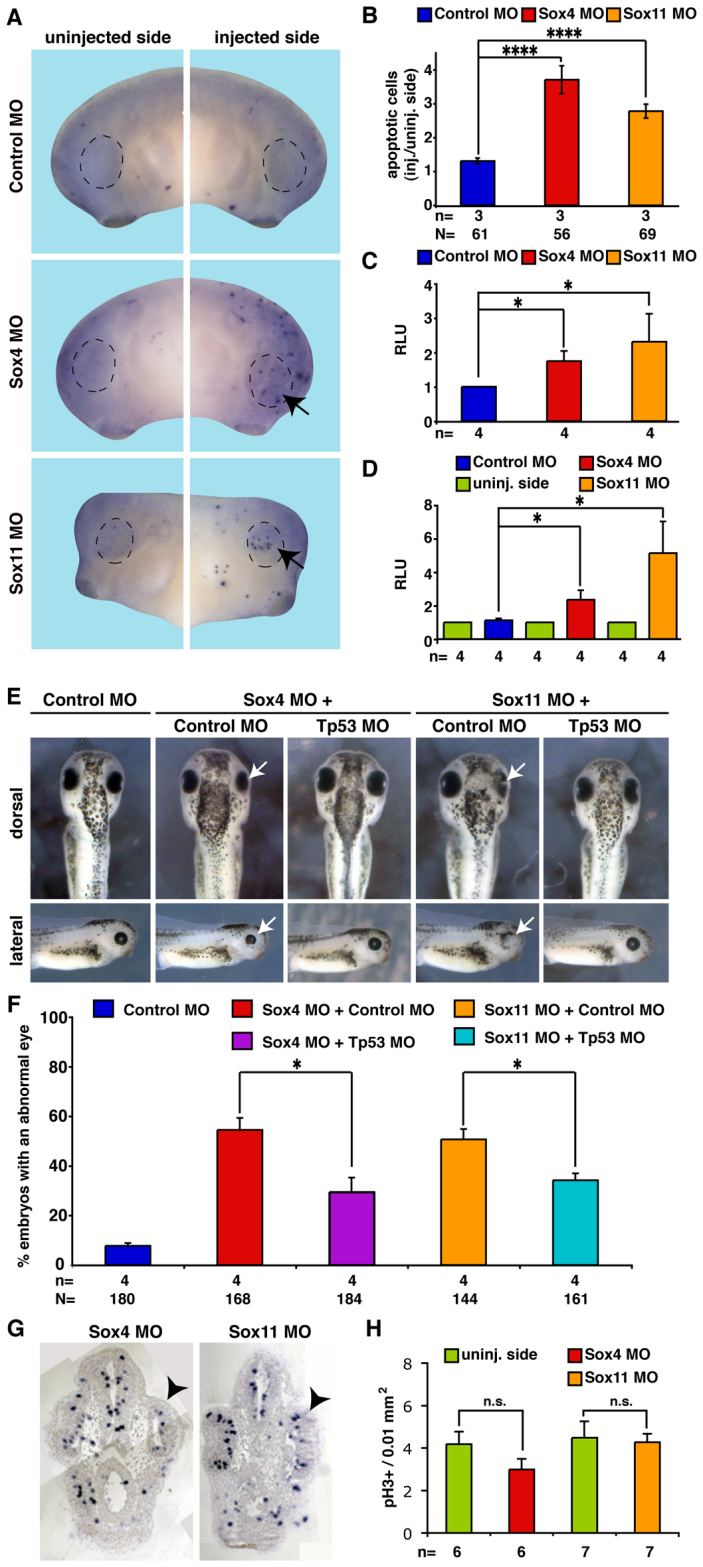
Loss of Sox4/11 function leads to cell apoptosis in the developing eye. **A:** TUNEL staining of Sox4 or Sox11 depleted embryos at stage 32. The areas where apoptotic cells have been counted are highlighted by a dotted circles and increased cell apoptosis was labeled by arrows. **B:** Quantitative representation of the TUNEL staining shown in A. n = number of independent experiments. **** P≤0.0001. **C:** Increased caspase 3/7 activity after loss of Sox4 or Sox11 at stage 32. Values represent relative light units (RLU) normalized to Control MO injected embryos. n = number of independent experiments. Error bars indicate standard error of the means (s.e.m.). **D:** Unilateral injection of 40 ng Sox4 or Sox11 MO led to increased caspases 3/7 activity while uninjected sides or Control MO injected embryos were not affected. Values represent relative light units (RLU) normalized to the uninjected side. n = number of independent experiments. Error bars indicate standard error of the means (s.e.m.). **E:** Increased cell apoptosis upon loss of Sox4 and Sox11 was rescued by tp53 inhibition. **F:** A quantitative representation of the results in E is given. n = number of independent experiments, N = number of injected embryos analyzed. **G:** Crosssections of Sox4 or Sox11-depleted embryos demonstrating mitotic cells (blue, pH3 staining). Black arrowheads point to the MO injected side. **H:** A quantitative representation of the data in F is given. Sox4 or Sox11 depletion had not significant (n.s.) effect on cell proliferation in the eye at stage 32. n = number of independent experiments. Error bars indicate standard error of the means (s.e.m.), n.s. not significant, * P≤0.05, **** P≤0.0001.

Next, we analyzed cell apoptosis using TUNEL staining on whole embryos (Fig. 7A,B) and a caspase 3/7 enzymatic activity assay using isolated heads (Fig. 7C). At stage 32, loss of Sox4 or Sox11 led to a significant increase in TUNEL positive cells in the developing eye region and an increased caspase 3/7 activity compared to uninjected or control MO injected embryo. This increase in apoptosis persisted until stage 41 (Fig. 7D). Sox4 has been described to be an anti-apoptotic factor by inhibiting Tp53 activity in hepatocellular carcinomas [18]. We therefore hypothesized that Sox MO injection may lead to an increased cell apoptosis in the *Xenopus* eye by activating Tp53. To test this hypothesis, we injected Sox4 or Sox11 MO together with Tp53 MO and observed a significant decrease in cell apoptosis in comparison to Sox MOs co-injected with Control MO (Fig. 7E,F).

To investigate cell proliferation, we made use of pH3 staining. Sox4 or Sox11 depletion did not significantly alter cell proliferation compared to uninjected or Control MO injected eyes at stages 32 (Fig. 7G,H) or 41 (data not shown).

In summary, Sox4 or Sox11 depletion resulted in a significant increase of Tp53-mediated cell apoptosis that might be the underlying cause for the smaller eyes observed.

## Discussion

In this study, we showed that (I) the HMG box transcription factors *sox4* and *sox11* are expressed in the developing *Xenopus* eye, (II) depletion of either Sox4 or Sox11 lead to severe malformations of the eye characterized by a decrease in size and disturbed retinal lamination, (III) loss of Sox4 or Sox11 does not affect neural induction, IV) Sox4 and Sox11 have no influence on the primary fate of most retinal cell types and (V) Sox4 or Sox11 down-regulation results in increased cellular apoptosis in the eye.

### *Sox4* and *sox11* Expression Across Species

We here provide a detailed description of the tissue specific expression of *sox4* and *sox11* during *Xenopus* embryogenesis and thereby extend earlier finding by others. The first cloning and maternal expression of *sox11* was described earlier by others [19]–[21]. The expression of *Sox4*, *Sox11,* and *Sox12* is highly conserved in mouse and chicken [7], [8], [22], in particular in the neural tube, the brain, the retina, the heart, and the kidney. A detailed expression of *Sox11* in the developing murine eye has been published [23]. Interestingly, the expression pattern of murine *Sox11* is quite similar to that of *sox4* as well as *sox11* during *Xenopus* eye development. Similar to our observation of *sox4* and *sox11* expression in the *Xenopus* eye, murine *Sox11* is expressed in the evaginating optic vesicle. Later in development, *Sox11* is strongly expressed in the retina, the lens fibers and the surface ectoderm. The RPE shows only a weak *Sox11* expression. In zebrafish, the two duplicated *sox11* genes, *sox11a* and *sox11b*, are also expressed in the developing eye and brain [24]. In contrast, *sox4a* is strongly expressed in the hindbrain and *sox4b* prominently expressed in the pancreas [25]. A second prominent expression site of *sox4* and *sox11* is the developing *Xenopus* heart. *Sox4* is expressed in particular in the forming outflow tract. This is in line with published data in the mouse since murine *Sox4* is also expressed in this region.

### Sox4 and Sox11 Function during Early Embryogenesis

In our study, we demonstrated that the tissue-specific depletion of either Sox4 or Sox11 in the anterior-neural tissue leads to severe malformations of the eye including smaller and deformed eyes with a disorganized retina lamination. These phenotypes fit to the specific *sox4* and *sox11* expression in the different *Xenopus* eye structures. Sox4 knock-out mice die at E14 because of severe cardiac defects [26]. Especially the formation of the outflow tract is compromised in these embryos resulting in circulatory failures. In addition, lymphocyte development is disturbed in these embryos. The formation of other organs including the eye was not investigated in this study. Since *sox4* is also expressed in the developing heart of *Xenopus*, it is certainly worthwhile to examine Sox4 function during *Xenopus* cardiac development by mesoderm-specific Sox4 MO injections.

Sox11 null mice die immediately after birth as a result of cardiac defects [27]. Moreover, these mice reveal problems during eye, spleen, lung, and bone development. The consequence of Sox11 depletion especially on murine eye development has been investigated in more detail [23]. Sox11 knock-out results in severe defects during the formation of the cornea epithelium and lens fibers. In addition, retinal folds develop and the lens stays in contact with the overlaying cornea epithelium (lens stalk).

One possible reason for the observed eye phenotype upon suppression of Sox4 and Sox11 function in *Xenopus* could be a perturbance of early neural or eye field induction and differentiation. In particular Sox11 gain-of-function in *Xenopus* animal cap cells results in neural induction [19]. Of note, loss of neither Sox4 nor Sox11 resulted in deficits in neural induction in our hands. We also investigated expression of *pax6* since Pax6 depletion in the mouse reveals a similar eye phenotype as loss of Sox4 in our study [28]. We detected a mild but significant change in expression of *pax6* and *rax*. This is in line with the observation of *Sox11* null mice that do not show a severe alteration of *Pax6* expression. Interestingly, *Sox11* is under the control of Pax6 [23] and it remains to be investigated whether this is also the case in *Xenopus*. In addition, proneural genes in the retina were not influenced upon loss of Sox4 or Sox11 function whereas ganglion cells differentiation was affected. These results are consistent with the observation that Sox4 and Sox11 act downstream of proneural genes but upstream of neuronal differentiation genes in the chick spinal cord [29].

### Sox4 and Sox11 in Cell Proliferation and Apoptosis

*SoxC* genes have been shown to be involved in cell proliferation and apoptosis [22], [23], [30]–[32]. During sympathetic nervous system development, depletion of SoxC proteins leads to a decrease in BrdU-positive cells [22]. In contrast, Sox4 and Sox11 double-deficient mice show only mild changes in cell proliferation compared to WT controls during spinal cord development [30]. In mice, Sox11 depleted lens epithelium reveal a reduction in cell proliferation at E10.5, whereas at E9 no obvious effect could be observed [23]. In contrast, we did not see any significant effect on proliferation in the *Xenopus* eye at different stages upon Sox4 or Sox11 depletion. Of note, these data do not exclude that in *Xenopus* Sox depletion might result in reduced cell proliferation at other developmental stages in the eye (or other organs).

The available results concerning Sox4 function in cell survival and apoptosis are contradicting. On the one hand, it has been shown that *SoxC* genes in general are required for cell survival of neural as well as mesenchymal progenitor cells through the Hippo pathway [31]. In line with this, Sox4 and Sox11 have been described as survival factors during the development of the spinal cord [30] and sympathetic nervous system [22]. Repression of tp53 activity was observed by gain of Sox4 function [18]. In contrast, Sox4 was described as a DNA damage sensor in lung carcinoma cells [32] promoting cell cycle arrest and apoptosis. In our study, we could show that loss of Sox4 or Sox11 function leads to an increase in cell apoptosis in the *Xenopus* eye at stages 32 and 41 and this might well contribute to the small eye phenotype. Moreover, this phenotype was restored by inhibiting Tp53 providing a potential mechanism in which Sox4 and Sox11 function as survival factors during *Xenopus laevis* eye development [18], [22], [30].

## Experimental Procedures

### *Xenopus Laevis* Embryos

*Xenopus* embryos were generated and cultured according to general protocols and staged according to others [33]. All procedures were performed according to the German animal use and care law (Tierschutzgesetz) and approved by the German state administration Baden-Württemberg (Regierungspräsidium Tübingen).

### Cloning and MO Injection

For loss of function experiments, morpholino oligonucleotides (MOs) were designed and ordered from GeneTools, LLC: Sox4 MO: 5′-ACCATTGCTGCTGCTGTTTAGCTAC-3′; Sox11 MO: 5′-TCTGCTCGCTGCACCATGGCTGTCA-3′ [10]; Tp53 MO: 5′- CCATGCCGGTCTCAGAGGAAGGTTC-3′ [34]. Sox4 MO is designed to bind both published *sox4* RNAs (sox4a: BC073494; sox4b: BC170171). For control injections, the standard control MO from GeneTools was used. All MOs were solved in DEPC-H_2_O and stored in aliquots at −20°C. For all experiments, 40 ng of the Sox MOs were injected unilaterally into one dorso-animal blastomere to target anterior neural tissue [35]. To analyze the role of Sox in apoptosis, 10 ng of Tp53 MO was coinjected with Sox MOs. 0.5 ng synthetic *gfp* RNA was coinjected as a lineage tracer in all experiments (see Fig. S2C). The uninjected side served as an additional, internal control. For rescue experiments, we used the full length human SOX4 construct of ImaGenes (clone #: IRAKp969B12110D) subcloned into pCS2+ vector [36]. To test the efficiency of the Sox4 MO, we cloned the *Xenopus* sox4 MO binding site (*xsox4-gfp*) and the corresponding region of the human construct (*hSOX4 MO-gfp*) in front of and in frame with the *gfp* open reading frame in pCS2+. 1 ng RNA of either fusion construct was injected together with either Control MO or Sox4 MO (Fig. S2B). *Sox11* rescue RNA was used as described before [10]. RNA concentrations injected were: 0.7 ng *sox11* and 0.5–1 ng *SOX4*.

### Whole Mount *In Situ* Hybridization

To analyze the spatiotemporal expression of *sox4* during *Xenopus laevis* embryogenesis, a full-length *sox4* cDNA was amplified with Phusion DNA-Polymerase (Biometra) from cDNA of stage 25 *Xenopus* embryos and ligated into the pSC-B vector (Stratagene). Primers used were: Sox4_f: 5′-TGC CCG GGG TGA CTG TAC TGC-3′; Sox4_r: 5′-TCA GTA GGT AAA TAC CAG GTT-3′. A DIG-labeled *antisense* RNA probe was generated by linearizing with ClaI (NEB) and *in vitro* transcription with T7 (Roche). Cloning and transcription of sox11 probe has been described earlier [10]. *Xenopus* embryos were fixed at different developmental stages with formaldehyde and WMISH analyzes were performed according to well established protocols [37]. For a more detailed analysis of gene expression, we performed sections of stained embryos with a thickness of 25 µm using a vibratome (Leica).

### PH3 and TUNEL Staining using Whole *Xenopus* Embryos

To detect cell proliferation as well as apoptosis in whole embryos upon Sox4 or Sox11 depletion, we performed pH3 *(phospho histone H3)* and TUNEL *(Terminal Deoxynucleotidyltransferase-mediated dUTP Nick End Labeling)* stainings according to standard protocols [37]–[39]. To investigate cell proliferation at stage 32, we made use of pH3 staining on whole embryos and counted pH3 positive cells on 7 µm plastic sections through the eye regions. We counted pH3+ cells in the eye on every third section (to prevent double counting of individual cells) and normalized the number of positive cells to the area covered by the eye.

### Caspase Assay

For the analysis of cell apoptosis at stage 32, Sox4, Sox11, or Control MO was bilaterally injected into both animal-dorsal blastomeres and the head region of embryos was dissected at stage 32. For the analysis of cell apoptosis at stage 41, the eyes of unilaterally injected embryos were dissected at stage 41 (injected as well as uninjected sides). Head regions or isolated eyes were homogenized in 70 µl PBS/Triton X-100 (0.05 M sodium phosphate, 0.9% saline, 0.1% Triton X-100, pH 7.4) and protein concentrations were determined by Bradford assay using BSA as standard. Caspase 3/7 assays were done using the Caspase 3/7 Glo Assay (Promega, Madison, WI, USA) as described [40].

### Statistics

*P*-values were calculated by a nonparametric Mann-Whitney rank sum test using GraphPad Prism 5 software.

## Supporting Information

Figure S1 **Transverse sections of an embryo at sate 34. A:** *Sox4* is expressed in the second (violet arrowhead) and first (red arrowhead) heart field lineage. **B:** *Sox4* is expressed in the vitelline veins. **C:** *Sox4* is expressed in the second heart field (black arrowhead) and the migrating neural crest cells (white arrow). **D:** *Sox4* is detectable in the forming outflow tract (black arrow) and the ventral aorta/aortic arch arteries (black arrowhead). **E:** *Sox4* is expressed in the mesocardium (me; black arrowhead) and the pericardium (p), and in the pericardial roof (pr), endocardium (e), and myocardium (m).
(PDF)

Figure S2 **Sox4 MO is specific. A:** Sox4 MO binding sites of *Xenopus* (*xsox4*) and the corresponding region of human SOX4 (*hSOX4*). **B:** Co-injection of xSox4 MO-gfp with control MO had no influence on gfp glowing. Sox4 MO blocked the translation of *gfp*. The human SOX4 binding site is not targeted by the Sox4 MO. **C:** The correct injection of Sox4 MO was controlled by *gfp* RNA coinjection. Dotted lines indicate the midline of the embryo.
(PDF)

Figure S3 **The eye phenotype after loss of Sox4 or Sox11 can be restored by both** ***SOX4*** **and** ***sox11*** **RNA.**
(PDF)
