# Supplementary figures and images for: sox4 And sox11 Function during Xenopus laevis Eye Development
Source: PLoS One. 2013 Jul 18;8(7):e69372. doi: 10.1371/journal.pone.0069372 (PMC3715537; doi:10.1371/journal.pone.0069372)

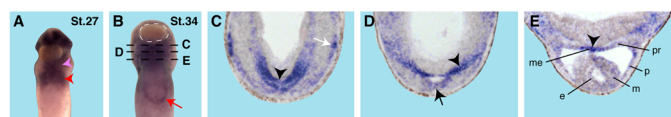

Cizelsky/Hempel, Figure S1

Supplement: Figure S1 — Transverse sections of an embryo at sate 34. A: Sox4 is expressed in the second (violet arrowhead) and first (red arrowhead) heart field lineage. B: Sox4 is expressed in the vitelline veins. C: Sox4 is expressed in the second heart field (black arrowhead) and the migrating neural crest cells (white arrow). D: Sox4 is detectable in the forming outflow tract (black arrow) and the ventral aorta/aortic arch arteries (black arrowhead). E: Sox4 is expressed in the mesocardium (me; black arrowhead) and the pericardium (p), and in the pericardial roof (pr), endocardium (e), and myocardium (m). (PDF) [file pone.0069372.s001.pdf]

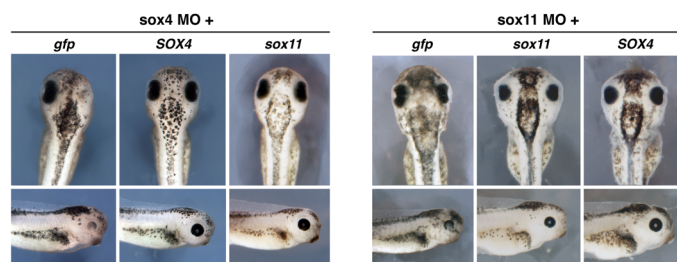

Cizelsky/Hempel et al., Figure S3

Supplement: Figure S3 — The eye phenotype after loss of Sox4 or Sox11 can be restored by both SOX4 and sox11 RNA. (PDF) [file pone.0069372.s003.pdf]
