# Supplementary material for: sox4 And sox11 Function during Xenopus laevis Eye Development
Source: PLoS One. 2013 Jul 18;8(7):e69372. doi: 10.1371/journal.pone.0069372 (PMC3715537; doi:10.1371/journal.pone.0069372)
Supplement: Figure S2 — Sox4 MO is specific. A: Sox4 MO binding sites of Xenopus (xsox4) and the corresponding region of human SOX4 (hSOX4). B: Co-injection of xSox4 MO-gfp with control MO had no influence on gfp glowing. Sox4 MO blocked the translation of gfp. The human SOX4 binding site is not targeted by the Sox4 MO. C: The correct injection of Sox4 MO was controlled by gfp RNA coinjection. Dotted lines indicate the midline of the embryo. (PDF) [file pone.0069372.s002.pdf]

A

sox4 MO binding sites:

xsox4 5'-GTAGCTAAACAGCAGCAGCAATGGT-3'
hSOX4 5'-ATCGATTCGAATTCGCCCTTATGGT-3'

green = start codon
red = different sequence

B **C**

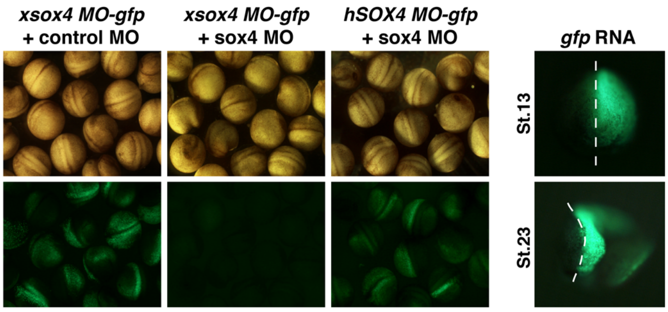

Cizelsky/Hempel et al., Figure S2
